# The immune epitope database (IEDB) 3.0

**DOI:** 10.1093/nar/gku938

**Published:** 2014-10-09

**Authors:** Randi Vita, James A. Overton, Jason A. Greenbaum, Julia Ponomarenko, Jason D. Clark, Jason R. Cantrell, Daniel K. Wheeler, Joseph L. Gabbard, Deborah Hix, Alessandro Sette, Bjoern Peters

**Affiliations:** 1 Division of Vaccine Discovery, La Jolla Institute for Allergy and Immunology, La Jolla, 9420 Athena Circle, CA 92037, USA; 2 Bioinformatics Core, La Jolla Institute for Allergy and Immunology, La Jolla, CA 92037, USA; 3 San Diego Supercomputer Center, University of California, San Diego, CA 92093, USA; 4 Leidos Health, LLC, San Diego, CA 92121, USA; 5 Grado Department of Industrial and Systems Engineering, Virginia Polytechnic Institute and State University, Blacksburg, VA 24061, USA

## Abstract

The IEDB, www.iedb.org, contains information on immune epitopes—the molecular targets of adaptive immune responses—curated from the published literature and submitted by National Institutes of Health funded epitope discovery efforts. From 2004 to 2012 the IEDB curation of journal articles published since 1960 has caught up to the present day, with >95% of relevant published literature manually curated amounting to more than 15 000 journal articles and more than 704 000 experiments to date. The revised curation target since 2012 has been to make recent research findings quickly available in the IEDB and thereby ensure that it continues to be an up-to-date resource. Having gathered a comprehensive dataset in the IEDB, a complete redesign of the query and reporting interface has been performed in the IEDB 3.0 release to improve how end users can access this information in an intuitive and biologically accurate manner. We here present this most recent release of the IEDB and describe the user testing procedures as well as the use of external ontologies that have enabled it.

## INTRODUCTION

The IEDB was established in 2004, and over the past 10 years our team has manually curated almost 16 000 published manuscripts and processed 200 direct submissions. As a result, detailed experimental data regarding more than 120 000 epitopes are now freely and easily accessible to the scientific community via most web browsers as a web-based interface. In addition, if one wishes to view 3D structural data using the Epitope Viewer application, Java 6 or 7 is required. The IEDB's primary curation focus is on data from scientific publications available in PubMed (1) focused on infectious diseases, allergy, autoimmunity and transplantation. Excluded from the primary scope are HIV-derived epitopes captured in the LANL database (www.hiv.lanl.gov/content/immunology) and cancer epitopes for which there is no resource currently available due to lack of support for such a resource by the National Institutes of Health. As an exception, all publications describing the 3D structure of an epitope in complex with its adaptive immune receptor or major histocompatibility complex (MHC) molecule are included regardless of origin of the epitope in order to provide a complete dataset of this particularly valuable type of information. Details describing the curation process put in place and followed by the curation team, including quality controls for accuracy and consistency, have been discussed previously (2).

The IEDB houses epitope-specific experimental assays. That is, every assay reflects the binding of an epitope-specific T cell receptor (TCR), antibody or MHC molecule to an experimentally tested antigen or epitope. The structure entered as the epitope is limited to the exact entity that was actually tested in the assay or was clearly deduced to be the epitope by the authors. In many cases this is not the minimal epitope and may not be limited to the contact residues of the epitope, but is rather a region containing the epitope. The fields of the IEDB describe the details of these experiments in great detail. First, the epitope structure is designated as either peptidic or non-peptidic. Peptidic epitopes are described by their linear amino acid sequence or as discontinuous amino acids by position within their source protein. Peptidic epitopes having 3D structural data are described by the residues found to contact the antibody, TCR or MHC molecule. Non-peptidic epitopes are manually curated by staff from the ChEBI team (3) who annotate the complete molecular structures using SMILES annotation. If the epitope was derived from a protein or a larger non-peptidic structure, these are also provided along with the organism in which these structures are found. For example, the linear epitope FEIKCTKPEACS is derived from the *Phleum pratense* (Timothy grass) protein Phl p 1. All experimental assays that characterize the epitope or its recognition by immune receptors are entered into the IEDB, including all negative data. For example, the FEIKCTKPEACS epitope has 15 assays curated from three different references. Details on the gender and age of the host who made the immune response and on the process that led to it (e.g. immunization, infection or other exposure) are also captured. Important aspects of antibodies are presented such as isotype, antibody name, clonality, etc. The processes and/or purification steps used to generate epitope-specific T cells, including *in vitro* restimulation steps are stored. Additionally, the type of assay used and every antigen studied are curated. As often as possible, external authoritative resources are utilized to provide standardized nomenclature and additional richness to the data. Examples include: use of NCBI Taxonomy (4) to describe organisms, GenBank (5) and UniProt (6) for proteins, ChEBI (3) for non-peptidic structures, the Ontology for Biomedical Investigations (OBI) (7) for assay types, Gazetteer (http://purl.bioontology.org/ontology/GAZ) for geographic location and the Human Disease Ontology (8) for diseases.

**Figure 1.**
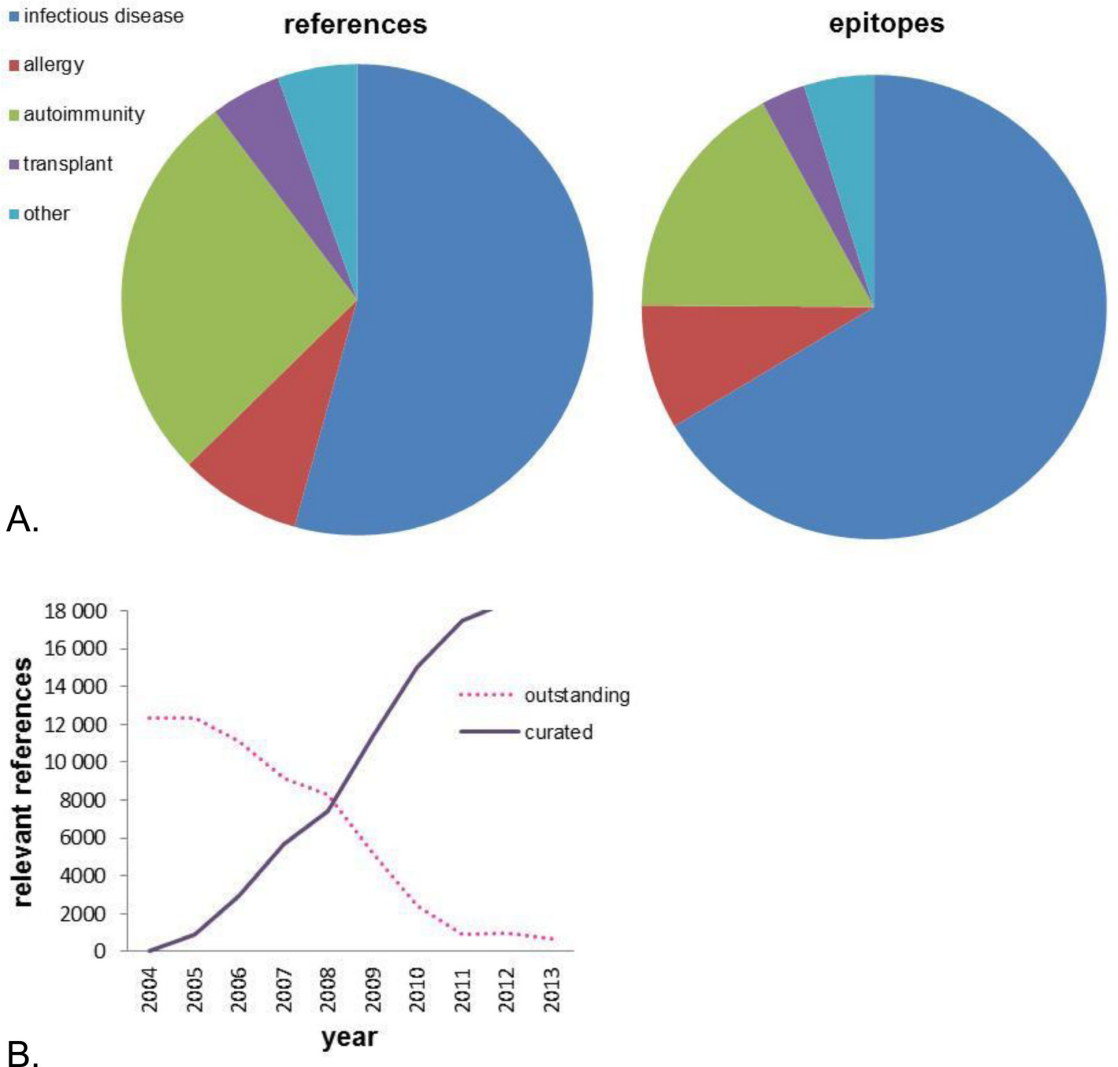
(**A**) The distribution of data in the IEDB by scientific field. (**B**) Curation of relevant references over time.

Figure 1A details the breakdown of the IEDB content currently as a function of the various main categories of epitopes and references. The category of infectious disease predominates at the level of numbers of epitopes and references. The category of ‘Other’ includes many references describing the 3D structure of an epitope with its adaptive immune receptor and, accordingly, these tend to have fewer epitopes. The IEDB reached the milestone of being current with >95% of relevant published literature at the end of 2012, as shown in Figure 1B. Since then, the IEDB curation team has been dedicated to remaining current with newly published literature meeting the goal of making new data available to users within eight weeks of publication. As the demands of curating the backlog of previous publication have now eased, the focus of the IEDB team has shifted to improving the user experience and providing new and useful functionalities.

## RESULTS

The driving force behind the IEDB 3.0 redesign has been user feedback accumulated since the 2.0 release of the IEDB in 2009 (2). Feedback was obtained from external immunological experts and database users through various channels, including *ad hoc* forums, help-desk requests/suggestions, a formal IEDB booth at major research conferences, and the annual IEDB user workshops. All feedback was compiled in 2013, in parallel with newly initiated mass appeals for feedback from web site users, contributors, developers and curators. Feedback from developers of related knowledge resources such as the PDB (9) was also solicited. In parallel, web site metrics were analyzed and analyses of queries made were performed to identify the most used search parameters. All gathered feedback was analyzed and redesign goals were set to incorporate as much feedback as possible and to make common queries easier to perform. Table 1 shows example feedback representative of the most commonly made requests. As shown, feedback was summarized into categories and actionable conclusions were drawn.

**Table 1.** Examples of the types of feedback gathered and the actions taken

Feedback	Sources	Category	Action
Allow one to further refine a query without having to use ‘back’ button	User observation, user help requests	New feature request	Added this ability
Provide downloadable graphics of immunome browser results	User help requests	New feature request	Added this ability
Provide pop-up hints where the user interface is not intuitive	User help requests	New feature request	Added this feature
Many links on home page rarely used	Web site metrics	Existing feature little used	Made lesser used links less prominent
Protein branch of the molecule tree better needs better nomenclature and synonyms	User observation, user help requests	Existing feature too complicated	Protein branch of the molecule tree was enhanced with these features
Make clearing selections easier	User observation	Existing feature too complicated	Simplified the interface
Analysis resource tools highly used, but hidden on home page	Web site metrics, user help requests	Existing feature difficult to find	These features were made more prominent
Add the ability to save queries	Workshop	New feature request	Not yet added, future
Confirm that the assay names are generally accepted in the immunological community	User observation	Existing feature too complicated	Not yet completed, future
Add cancer epitopes	Workshop, IEDB booth	New scope request	Will not do, out of scope

The two main repeated requests made for the search interface were the ability to refine search results and to ensure that the restriction of the original search would not get lost when drilling down into search results. To enable this, a completely new approach for search was formulated. This plan is analogous to the search on a travel web site, whereby a typical user first enters very simple key search parameters, such as where they wish to travel from and to and on what dates. Once the results meeting these criteria are displayed, the user has the ability to further limit the results based upon additional parameters, for example, further limiting to only nonstop flights, and then to decide upon a specific flight choice. Following this model, the home page was redesigned to present users with the most commonly used search parameters on the home page followed by a results summary page that adds additional filters allowing further refinement of the dataset. In parallel, we sought to improve the presentation and utility of the data displayed on the web site.

**Figure 2.**
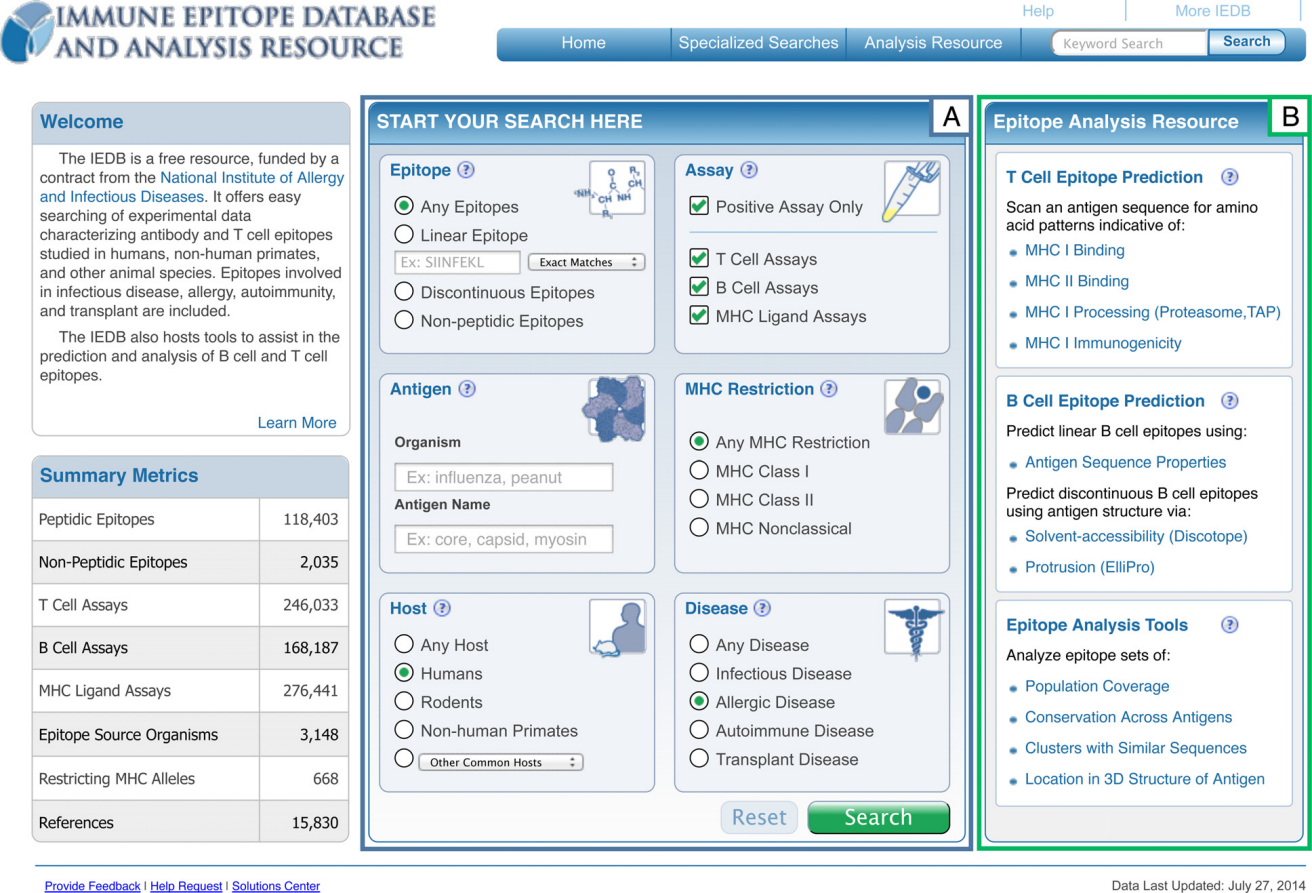
The IEDB 3.0 home page has the most commonly used search parameters centered on the page, shown in box (**A**), with the highly used analysis tools made more prominent, shown in box (**B**).

The form-based travel web site model was favored over the even simpler ‘Google-style’ interface because the IEDB houses well-structured data and the same search term can be found in different database fields, leading to unintuitive results if all are returned. For example, ‘human’ could refer to the host mounting the immune response or the source protein of the epitope, such as when human insulin is tested for immunogenicity in rats. At the same time, it is crucial to not overwhelm the users with too many options for their search as the IEDB contains more than 400 searchable fields, most of which are included in the ‘specialized’ search of the IEDB that only relatively few expert users are employing. To avoid overwhelming users with this large number of potential parameters, the home page search fields were limited to only include the most commonly used search parameters which are now placed prominently in the center of the home page. As shown in box A of Figure 2, the home page search fields are organized as discrete search sections, typical of the most common queries performed on the IEDB's site, with explanatory icons and help links embedded into the page. New icons were designed to highlight the main search components used for epitope related data. Icons were chosen based on a survey of scientists asked to identify the most relevant icon from a set to represent each major search parameter. These icons are similar to ones for hotel, airfare, or car rental on a travel web site, distinguishing the major types of searches possible. These search sections also serve to restrict search terms to specific database fields and help guide the user as to the types of data that the IEDB contains. For example, in the ‘Host’ section, a variety of hosts including humans, rodents, non-human primates, and an additional nine commonly studied species are presented.

**Figure 3.**
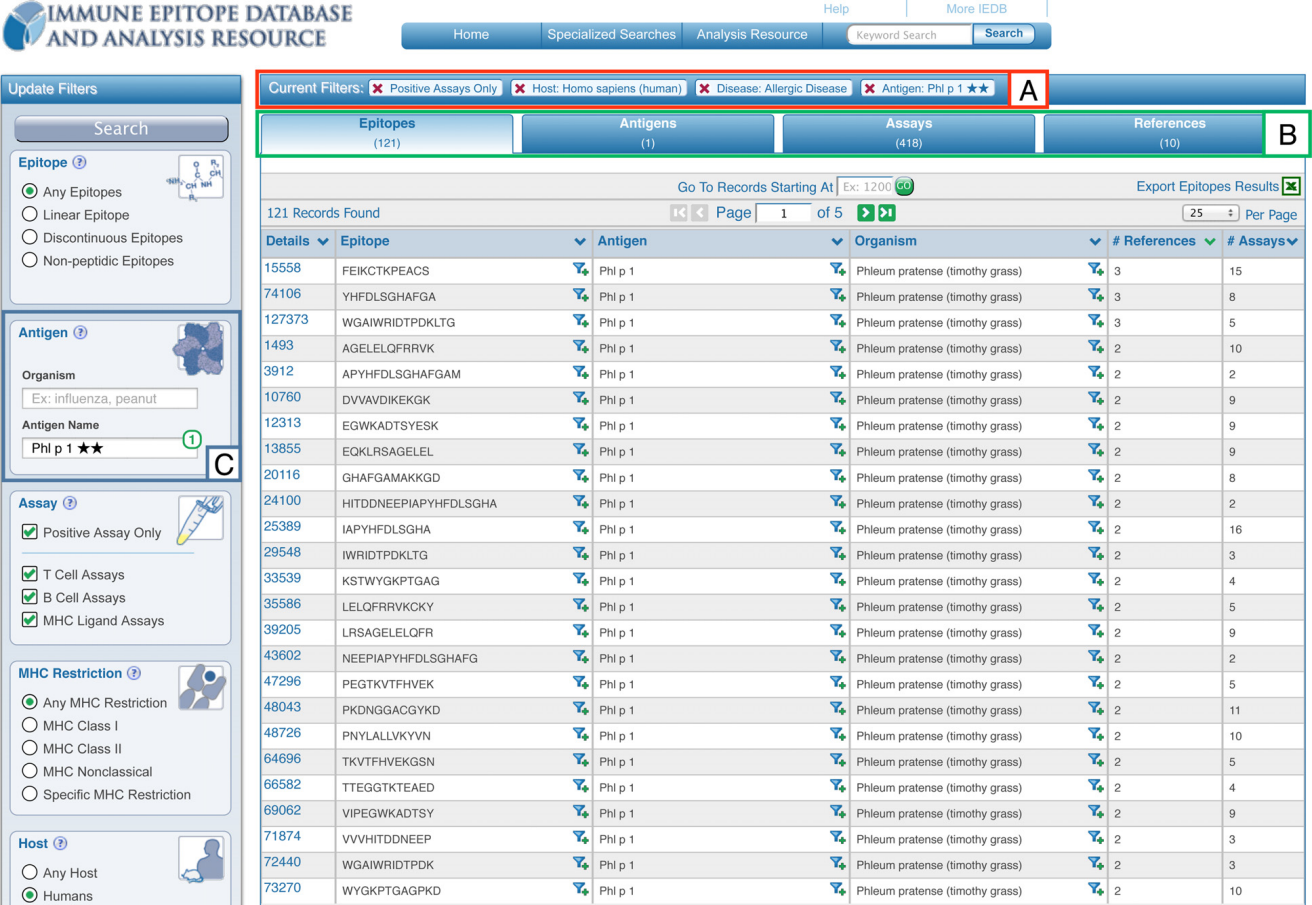
New results presentation format shows current search filters in box (**A**), counts returned per data type in box (**B)** and the new left search panel allowing for continued refinement or editing of one's query, such as by the epitope source, in box (**C)**.

Once a query such as the one populated in Figure 2 has been executed, the search results are presented on a new page with the current search filters displayed at the top of the results table (Figure 3, box A). Any filter can be removed by a single click on the ‘X’ next to each parameter. The amount of data present within each of the result set types of Epitopes, Antigens, Assays and References are conveyed by counts and displayed as tabs that allow the user to easily navigate between them (Figure 3, box B). As shown in Figure 3 box C, a search panel added to the left side of the page allows the current result set to be further refined by adding search parameters or to run a new query entirely. These search panels contain the functionality present on the home page plus several additional search features, some of which were previously only present in the IEDB 2.0 ‘Advanced Search’, such as the ‘Assay Types.’ We plan to continuously monitor the usage of each search parameter to identify additional fields that should be added to or removed from the search panel on the results page.

In addition to the query interface, the presentation of the results has been modified as well. Query results are grouped in four tabs: Epitopes, Antigens, Assays and References that match the current search criteria (Figure 3, box B). These different units of information reflect that some users want to utilize the IEDB as, for example, a way to explore the literature (on the reference tab), while others want to see which specific proteins in an organism have been studied for immune reactivity (on the antigen tab). The amount of data hosted in the IEDB has grown dramatically in the last few years, so that typical queries retrieve a very large number of epitopes. To make sure the most relevant epitopes are immediately visible, results are now sorted by how much information is available, such as the number of references with relevant data, as shown in Figure 3, rather than alphabetically, as was previously done. In addition to the left search panel, users can click on an epitope structure or its source to further narrow the result, using a new ‘filter’ icon present in the results table. Another noteworthy enhancement in the IEDB 3.0 is a new ‘Antigen’ tab which displays all epitopes that belong to the same antigen in one row. The Antigen table also provides information on how often epitopes from each antigen were studied with counts for number of epitopes, assays and references relevant to each antigen. Users may further narrow their results to a single antigen using the ‘filter’ icon present in the results table, or use another updated feature, the ‘Immunome Browser,’ which is discussed below.

Other search features that have been redesigned throughout are the ‘Finder’ elements, most notably the Molecule Finder in the antigen search panel. Accessed as shown in Figure 3, box C, this finder provides a hierarchical organization of proteins that allows narrowing the search to epitopes derived from a specific antigen, such as the common allergen Phl p 1. Navigation of proteins within the Molecule Finder was identified as being overly difficult based on user feedback, so it was redesigned to simplify this process. As each protein is derived from an organism, this redesign process began with a major simplification of the organism tree.

**Figure 4.**
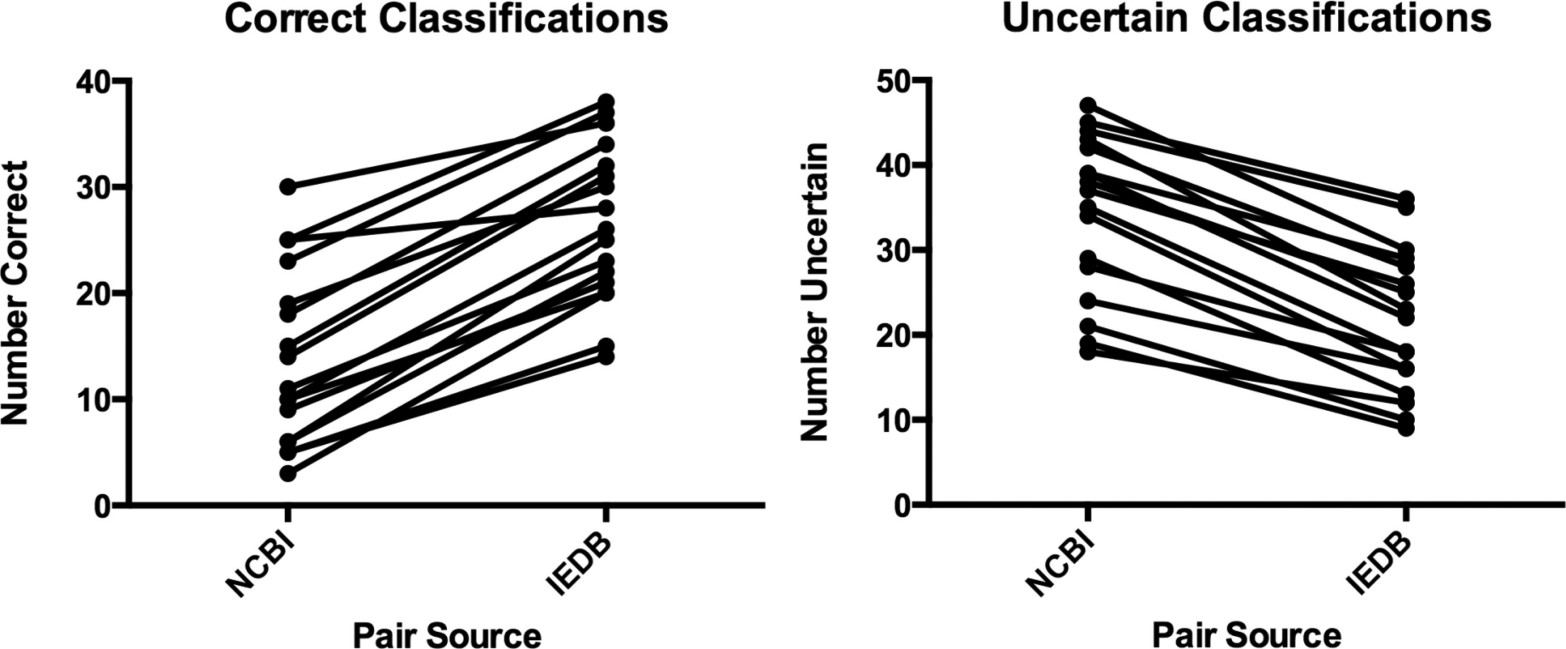
Comparison of classifications of pairs between NCBI Taxonomy and IEDB organism tree. Left: correct classifications. Right: uncertain classifications. The Wilcoxon signed rank test shows that both results are statistically significant with a *P*-value of <0.0001.

The organism tree is based on the NCBI Taxonomy (4), which contains hundreds of thousands of taxa in a hierarchy up to 39 nodes deep. The new organism tree uses a carefully selected subset of the full NCBI Taxonomy that covers all the taxa used in the IEDB, and reduces the depth of the hierarchy for easier navigation by immunologists. We tested the ability of users to correctly classify organisms using either the original NCBI Taxonomy or the revised IEDB organism tree and found that more correct classifications and more certainty in the choices made was obtained using the IEDB organism tree (Figure 4). This was accomplished by presenting users with 50 pairs of a species label with a higher taxon label. The pairs were randomly selected, half from the NCBI Taxonomy and half from the IEDB organism tree. In answer to the question ‘Is [species] a [higher taxon]?’ (e.g. ‘Is “*Narcine timlei* (blackspotted numbfish)” a “Serpent (snake)”?’) users could choose ‘true,’ ‘false’ or ‘I would have to guess.’ The users had a range of taxonomic knowledge, but all made more correct classifications and showed more certainty when classifying pairs from the IEDB organism tree as compared to the NCBI Taxonomy.

**Figure 5.**
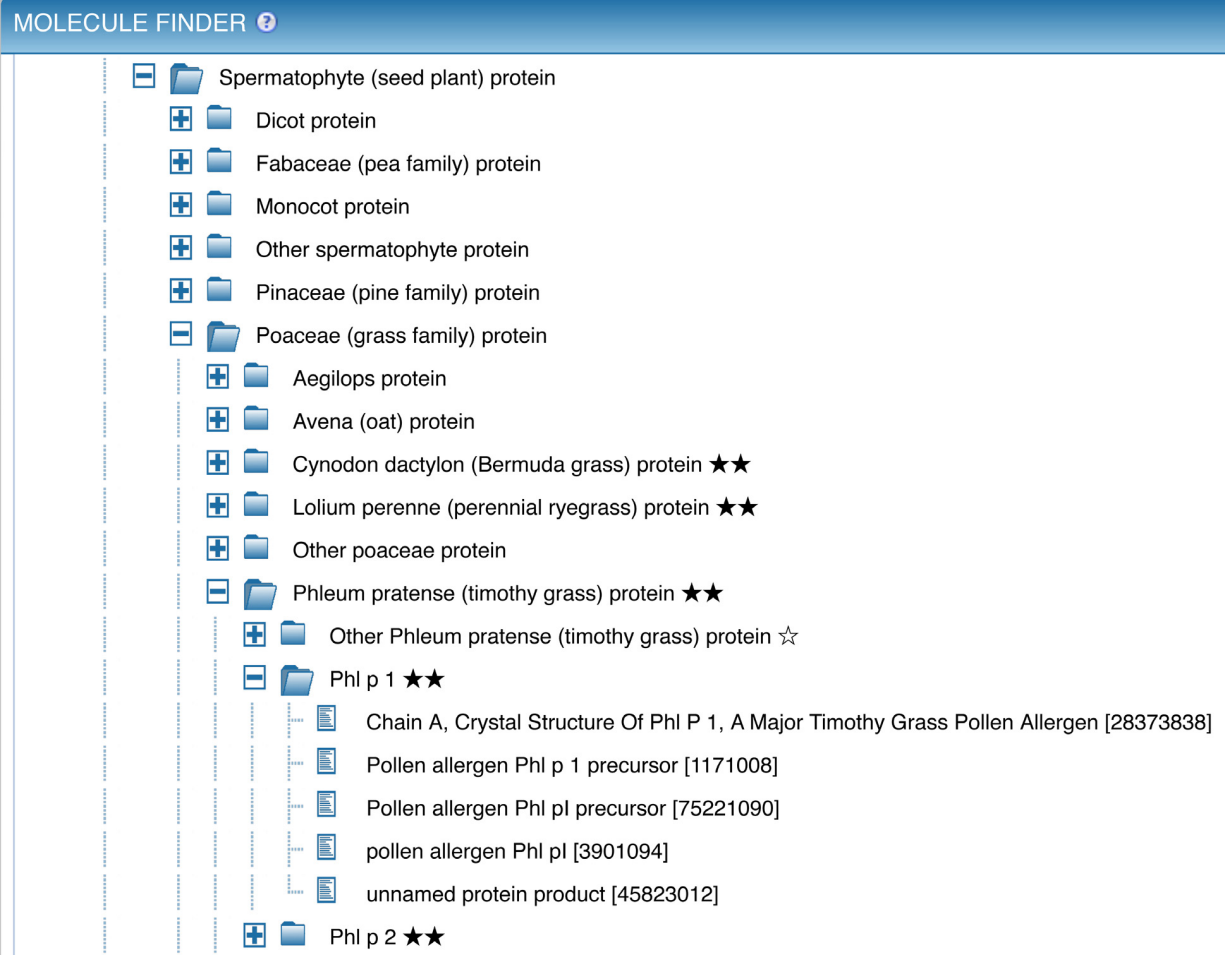
The Molecule Finder provides a hierarchical organization of proteins that allows narrowing the search to epitopes derived from a specific antigen, such as the common allergen Phl p 1. The reference proteome protein ‘Phl p 1’ is the parent of five individual GenPept entries for this protein from Timothy grass.

The Molecule Finder has two top-level branches for peptidic and non-peptidic epitopes. Non-peptidic epitopes are assigned to sources in ChEBI (3) and displayed using the ChEBI hierarchy. Peptidic epitopes derived from proteins occurring in nature have their specific source protein identified by GenPept (5) entries. The variety of distinct sequences represented in GenPept (e.g. the five versions of Phl p I shown in Figure 5) is necessary and reflective of the heterogeneity of proteins within individual species; however, the large number of entries and lack of standardized nomenclature previously overwhelmed users, and made it difficult to obtain all epitopes belonging to a single antigen.

To simplify the representation of proteins within the IEDB we now use UniProt (6) reference proteomes for each species (whenever possible) and use them as parent nodes under which GenPept entries are distributed based on sequence similarity. The use of reference proteomes ensures that each antigen is present just once in the tree, that a consistent nomenclature is utilized, and that additional information such as synonyms and protein classifications can be utilized. If no UniProt reference proteome was available, we constructed alternative reference proteomes in a semi-automated fashion. These are meant to serve as placeholders until the corresponding reference proteomes become available from UniProt. The quality and completeness of proteomes are indicated to the users using a system of stars. As shown in Figure 5, three stars indicate a UniProt reference proteome, two stars indicate a complete UniProt proteome that has not yet been reviewed, and a single star is used for proteins that are not part of a proteome.

**Figure 6.**
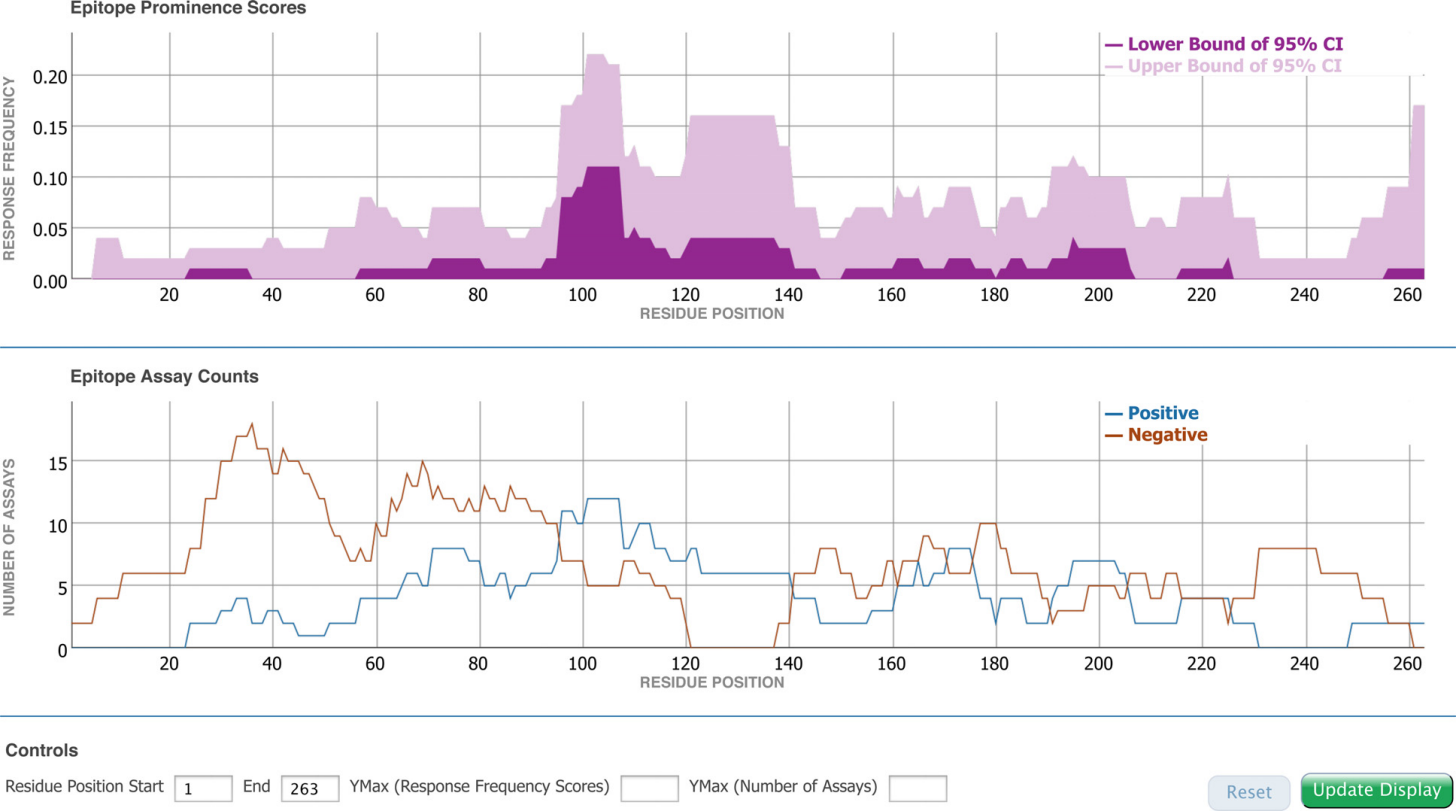
Immunome Browser plots for the epitopes from the Timothy grass allergen Phl p 1 recognized in the human T cell response.

The improvements made to the Molecule Finder benefit the Immunome Browser, which can utilize the reference proteomes as mapping targets. Previously available as an on-demand visualization tool in the IEDB (10), the Immunome Browser has now been tightly integrated within the antigen tab, redesigned and enhanced based on user feedback. Conceptually, the Immunome Browser is the first analytical tool integrated into the IEDB database, as it does not simply display information as stored in the database, but maps epitopes onto a reference antigen. Similar to the now commonly used genome browsers, this allows for the aggregation of information derived from different sources and their display in a common reference. On the antigen tab, the Immunome Browser can now be used to immediately visualize linear peptidic epitopes retrieved by a query along the length of the parent antigen based on sequence similarity. This displays how often each protein region has been studied in immune assays and in how many assays the immune response was positive or negative. Figure 6 shows the Immunome Browser output for the epitopes from the Timothy grass allergen Phl p 1 recognized in the human T cell response. The upper plot renders the lower and upper bounds of the 95% confidence interval of the response frequency for each target protein position, averaged over all epitopes mapped to that position and calculated as the number of positively responded subjects (or individuals in this case) relative to the total number of those tested. The bottom plot shows the number of positive and negative assays averaged over epitopes mapped to each position in the protein sequence. A table below the graphs (not shown) presents results for each epitope and each protein position in a tabular format that can be saved, along with the graph images, for further analysis and publication. The user can interactively zoom in and out the plots to a specific protein region and the table will update accordingly.

In addition to the most common application of mapping epitopes from different protein variants to a common reference antigen, the Immunome Browser can also map any set of epitopes retrieved by a query to any user-specified protein or protein set. This enables analyses such as which viral epitopes have homologs in the human proteome. Controls to change the mapping criteria and the target protein/proteome to which the epitopes are to be mapped are also provided.

It is worth noting that the entire process of redesigning the IEDB was performed under the expert guidance of several consultants, including two usability experts and a graphic artist. The usability experts compared the user interface design to established design guidelines and successful extant interaction metaphors (11). This identifies critical usability problems early in the development cycle, so that these design issues can be addressed as part of the iterative design process (12). Iterative ‘design, implement and evaluate’ cycles were used as the IEDB user interface continually evolved. Feedback from the graphic artist and the usability experts was implemented regarding the color scheme, font and style with changes being made across all aspects of the graphics in order to update the general look of the web site, make it self-consistent and visually direct the users toward the most applicable features. The design, placement and functionality of links, search boxes, radio buttons and drop down lists were discussed with users and experts and each was redesigned. For example, the search panels on the results page filters were logically grouped and organized along the left-hand side of the page to be more consistent with commercial web retailer filtering metaphors, which have become de facto ‘standards.’

## CONCLUSION

After catching up on the curation of in-scope journal articles from the past, the focus of IEDB development for the 3.0 release has shifted toward improving query and reporting interfaces. The goal of this release was to provide intuitive ways to extract biologically accurate information from the large amounts of data now stored in the IEDB. We have here described the main new elements of the 3.0 release, all of which were motivated by user feedback gathered over the years. We believe that such development focusing on the usability of the web site is equally important to the introduction of new capabilities which—while often more exciting to implement from a web site developer's perspective—have little value if they are not actually utilized by the user community.
